# Dr. Geo. T. Brown

**Published:** 1892-07

**Authors:** 


Dr. Geo. T. Brown, of Atlanta, is now in New York, at the Post-Graduate Medical School, pursuing a special course in the eye, ear and throat.

